# Gleanings from Our Exchanges

**Published:** 1874-12-15

**Authors:** 


KLEIN ON THE ANATOMICAL CHANGES IN TYPHOID FEVER (*Medical Times and Gazette*, Oct.24, 1874).—Dr. Klein, of the Brown Institution, has lately made some interesting observations on the above subject. Sections of the hardened ileum of typhoid patients show, according to him, that an active absorption of peculiar organisms goes on in the mucous membrane of, and especially around, the Peyer's patches. These organisms are carried thence into the lymph-canals and the vessels of the mucous membrane.

In the earliest case which he examined, where death had occurred on the seventh day after the first appearance of headache, the crypts of Lieberkuhn were found to contain peculiar greenish-brown spheroidal corpuscles of very variable size, the largest twice or three times as big as a human red blood-corpuscle, the small ones only half or a quarter as large. When the bodies lie closely grouped together, as is generally the case, they appear of a dark olive-green color; and the corpuscles at the edge of such masses, or where they are completely isolated, exhibit transitional forms, due to incomplete subdivision. Similar corpuscles are found in the tissue of the mucous membrane, where they appear to be contained in the lymphoid cells of the adenoid tissue. The minute veins, and also some of the lymphatic vessels, contain large numbers of them, and in the former they subdivide rapidly, so as to form greenish-yellow granular micrococci, arranged in groups of two or four, as well as in rings and other figures. The micrococci have their origin in a mycelium whose filaments are branched and apparently smooth, and of a greenish-yellow color. These organisms occur not only in the neighborhood of Peyer's patches, which are moderately swollen, but also in parts of the mucous membrane which to the naked eye show no alteration except slight general swelling, although, microscopically, the follicles of the patches in one case were found to have undergone the following changes: The central part of the follicle, especially where it lies in the submucous tissue, was converted into a spongy substance by the formation of spaces around its blood-vessels, their wall consisting of the adenoid tissue with which the latter are sheathed. The lymphoid cells of this tissue were converted into large granular bodies containing two to five or even more nuclei, which greatly resembled the nuclei of the endothelial cells. In several of the follicles true giant cells were seen.

In a later stage (twelfth day) the mucous membrane itself showed somewhat similar changes, and the multinuclear lymphoid cells were found in its venules and in those of the submucous tissue, as well as in the lymphatics of the latter. Dr. Klein is unable at present to give a decided opinion whether the above alterations are directly dependent on the presence of the micrococci, or whether they must be considered as secondary to changes in the vascular system. The passage of micrococci inwards from the free surface of the intestine can be traced through the epithelium into the substance of the mucous membrane, and especially towards the crypts of Lieberkuhn; and this occurs in parts which are some distance from the swollen Peyer's patches, and which appear nearly or quite unaltered to the naked eye.—*Boston Medical and Surgical Journal.*

---

SCARLATINAL WAVES.—*The British Medical Journal* of October 17, 1874, in an editorial on this subject, states that the scarlatinal wave for a year is nearly always at its lowest point in spring, and at its highest late in autumn, usually in the months of April and November. This may be called the annual wave, and varies but little in its course, whether the disease be epidemic or not. An examination of the deaths in the metropolis (London), recorded during thirty-two years, shows that the *lowest* point in each year was reached, on fifteen occasions, between the tenth and fifteenth weeks, and in nine others between the fifteenth and twentieth weeks; that the *highest* point in each year was reached, on sixteen occasions, between the fortieth and forty-fifth weeks, and on thirteen between the forty-fifth and fiftieth weeks. The total mortality in the thirty-two years, during the five weeks which are included between the beginning of the eleventh and the end of the fifteenth week, amounted to 5,204 deaths, whilst during five weeks which are included between the beginning of the fortieth and the end of the forty-fourth week in the same year, the deaths amounted to no less than 12,172.

Another wave, which may be called the *periodic*, may be represented by a line connecting together the mortality from the disease in each year, and indicates the years in which it is epidemic or non-epidemic. An examination of the mortality of each of the thirty-four years ending Dec. 31, 1873, shows that the disease was epidemic in 1840, 1844, 1848, 1852, 1854, 1858–59, 1862–64, and 1868–70; whilst the smallest mortality occurred in 1841, 1846, 1851, 1857, 1861, 1867, and 1873. It is, therefore, evident that the curve of the descending is much more gradual than that of the ascending wave, as the epidemic takes a longer time to subside than to rise again. The almost uniform recurrence of the disease as an epidemic, after three years of comparatively small mortality, is very noticeable in the figures just quoted.

What are the causes of this periodical increase in the height of the scarlatinal wave? Does it arise from seasonal influences, or other causes at present unknown? To this we can only reply, at present, that the careful comparisons made by Dr. Tripe in 1848, and by Dr. Richardson some years afterwards, show that a temperature below 44-6° Fahr. corresponds with the spread of scarlet fever, whilst a temperature above that point is coincident with an increase in the mortality; also, that the greatest mortality in the year occurs when the temperature ranges between 49-6° and 56-9°, but that the movement in the mortality does not occur in the same ratio with the increase in the temperature. This latter conclusion might have been expected from the comparative regularity with which the disease assumes an epidemic form every four years, whilst there are not, so far as we know, any corresponding sequences in any of the atmospheric phenomena. There is one important consideration respecting scarlatina, as well as small-pox and other eruptive diseases which occur ordinarily only once in a person's life, which must not be forgotten, viz., that in the interval between one epidemic and another a number of children are born who are susceptible to the disease from not having had it, and the epidemic may chiefly take its origin by the disease occurring in localities where there are many children unprotected, and thus spread rapidly to persons in the immediate vicinity. This can hardly explain its periodicity, although it accounts for the greater number of cases when the outbreak occurs.—*Boston Medical and Surgical Journal.*

---

PROF. ESMARCH recently attended a meeting of the Clinical Society of London, and gave a very interesting account of his method of controlling hæmorrhage, together with some reports of cases in which he had seen gratifying success attend its uses. Out of three hundred cases in which the method has been employed by him, no evil result has followed in any one. His cases had done better, he said, than those treated antiseptically, and with the ordinary means of controlling hæmorrhage.

---

INJECTIONS OF COD-LIVER OIL FOR ASCARIDES.—The *Journal des Connaissances Medicales* publishes a communication from Dr. Szerleki, of Mulhouse, on a case of irritation of the anus and adjoining parts, which was very greatly relieved by injecting an ounce of cod-liver oil into the rectum.—*The Obstetrical Journal.*

